# IRF8 Governs Expression of Genes Involved in Innate and Adaptive Immunity in Human and Mouse Germinal Center B Cells

**DOI:** 10.1371/journal.pone.0027384

**Published:** 2011-11-11

**Authors:** Dong-Mi Shin, Chang-Hoon Lee, Herbert C. Morse

**Affiliations:** Laboratory of Immunopathology, National Institute of Allergy and Infectious Diseases, National Institutes of Health, Rockville, Maryland, United States of America; National Institute of Environmental Health Sciences, United States of America

## Abstract

IRF8 (Interferon Regulatory Factor 8) is a transcription factor expressed throughout B cell differentiation except for mature plasma cells. Previous studies showed it is part of the transcriptional network governing B cell specification and commitment in the bone marrow, regulates the distribution of mature B cells into the splenic follicular and marginal zone compartments, and is expressed at highest levels in germinal center (GC) B cells. Here, we investigated the transcriptional programs and signaling pathways affected by IRF8 in human and mouse GC B cells as defined by ChIP-chip analyses and transcriptional profiling. We show that IRF8 binds a large number of genes by targeting two distinct motifs, half of which are also targeted by PU.1. Over 70% of the binding sites localized to proximal and distal promoter regions with ∼25% being intragenic. There was significant enrichment among targeted genes for those involved in innate and adaptive immunity with over 30% previously defined as interferon stimulated genes. We also showed that IRF8 target genes contributes to multiple aspects of the biology of mature B cells including critical components of the molecular crosstalk among GC B cells, T follicular helper cells, and follicular dendritic cells.

## Introduction

IRF8, one of nine members of the IRF family of transcription factors, functions in modulating immune responses and as a central element in the IFN signaling cascade. The gene is constitutively expressed in macrophages where it has been identified at the promoter regions of a large number of genes critical to macrophage differentiation and function [1]–[5] **.** Macrophages of mice deficient in IRF8 due to a conventional gene knockout (KO) [6] or a spontaneous mutation (IRF8^R294C^) in BXH2 mice [7] remain immature and are susceptible to a variety of infectious agents [8]–[10].

Studies of IRF8-deficient mice also identified critical roles in dendritic cell (DC) development and function. IRF8 KO mice lack plasmacytoid DCs (pDC) and CD11c^+^CD8α^+^ DCs [11], [12]; however, R294C mutant mice lack only CD8α^+^ DCs indicating that distinct IRF8-dependent mechanisms mediate the development of these two DC subsets.

Early on, it was shown that IRF8 is constitutively expressed by normal mouse B cells and lymphoma cell lines with features of pro-B and pre-B cells but not by plasmacytomas, tumors of mature plasma cells [13]. The contributions of IRF8 to early B cell development in mice were found to include involvement in the transcriptional networks controlling B cell lineage specification, commitment and differentiation in bone marrow [14] with regulation of the pre-B to B cell transition being dependent on heterodimerization of IRF8 with another IRF family member, IRF4 [15]. The recent development of IRF8 conditional knockout mice made it possible to determine B cell lineage-specific effects of IRF8 deficiency [16]. These studies showed that IRF8 normally acts to control the sizes of both the splenic marginal zone and follicular B cell populations while having little effect on responses to immunization with T-dependent or T-independent antigens.

Additional studies showed that among mouse and human B lineage cells IRF8 is expressed at the highest levels in germinal center (GC) B cells and lymphomas of GC origin but is extinguished in terminally differentiated plasma cells and plasma cell neoplasms [17], [18]. IRF8 was shown to contribute to the GC reaction by modulating the expression of BCL6, AID and MDM2 [17], [19]. Although some of the transcriptional programs and cellular pathways that mediate IRF8 effects in myeloid and DCs have been worked out in great detail [4], [10], [20], [21], much less is known about these aspects of IRF8 in B cell biology. The present studies were directed at broadening our understandings of these processes utilizing i) ChIP-chip analyses to identify IRF8 targets in human and mouse lymphoma cell lines of GC origin, and ii) gene expression profiling of a lymphoma cell line of GC origin and IRF8 siRNA knockdown subclones.

## Results and Discussion

### Identification of IRF8 targets in cell lines derived from human lymphomas of GC origin

**Figure 1:**
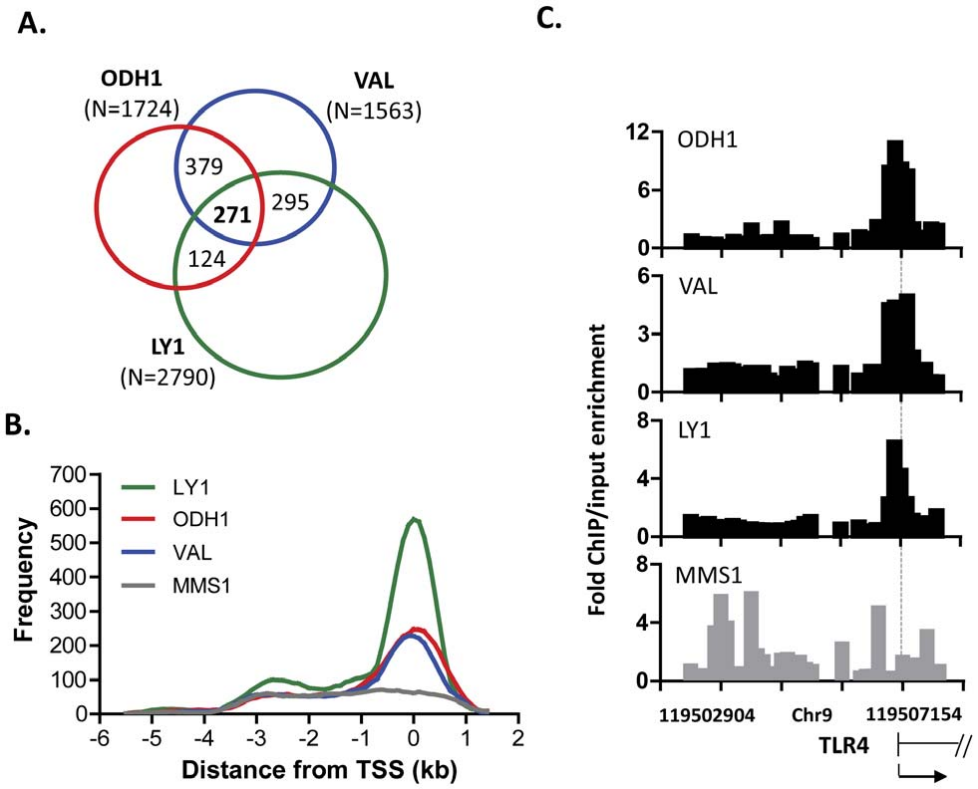
Identification of IRF8 targets in human cell lines of GC B cell origin. Labeled IRF8 ChIP samples and input samples from the three human cell lines of GC B cell origin were applied to Nimblegen HG18 385 k arrays and peak signals were analyzed by sliding window algorithm with threshold of FDR<0.01. (A) Venn diagram of IRF8 targets in three cell lines. Numbers in parentheses indicate the numbers of targets identified in each line. Internal numbers indicate targets common to two or all three lines. (B) Distribution of IRF8-bound sites relative to the transcription start site (TSS) of target genes. (C) Representative IRF8 binding from ChIP-chip in four cell lines. Binding of IRF8 to the TLR4 promoter is shown as an example. Fold change is calculated from relative fold enrichment of IRF8 ChIP signal to input signal. Dashed line shows TSS.

To identify direct transcriptional targets for IRF8 in human GC B cells, we hybridized IRF8-bound chromatin obtained by ChIP from three cell lines of GC origin (ODH1, VAL and LY1) to Nimblegen promoter tiling arrays consisting of probes covering 3.5 kb upstream to 0.75 kb downstream of transcriptional start sites (TSS); a multiple myeloma cell line (MMS1) with very little or no expression of IRF8 served as a negative control. The number of genes identified as IRF8-bound in the three GC lines were 1,563 for VAL, 1,724 for ODH1 and 2790 for LY1 with 271 genes being common to all three lines (Figure 1A; Table S2). These binding sites were identified by applying the false discovery rate (FDR)<0.01 to IRF8-specific enriched peaks detected by the sliding window method.

Mapping of probes targeted by IRF8 to the human genome showed that the great majority fell within well-defined peaks located from 1 kb 3′ to 1 kb 5′ from the TSS of involved genes (Figure 1B). In contrast no significant peaks were observed with material prepared from the negative control cell line, MMS1, although a low frequency of targets extended from −4 kb to +1 kb. While target sites identified in ODH1 and VAL lying outside this interval were indistinguishable from the pattern for MMS1, a small subset of targets lying 2 kb to 3 kb upstream of the TSS were seen for LY1 (Figure 1B).

**Figure 2:**
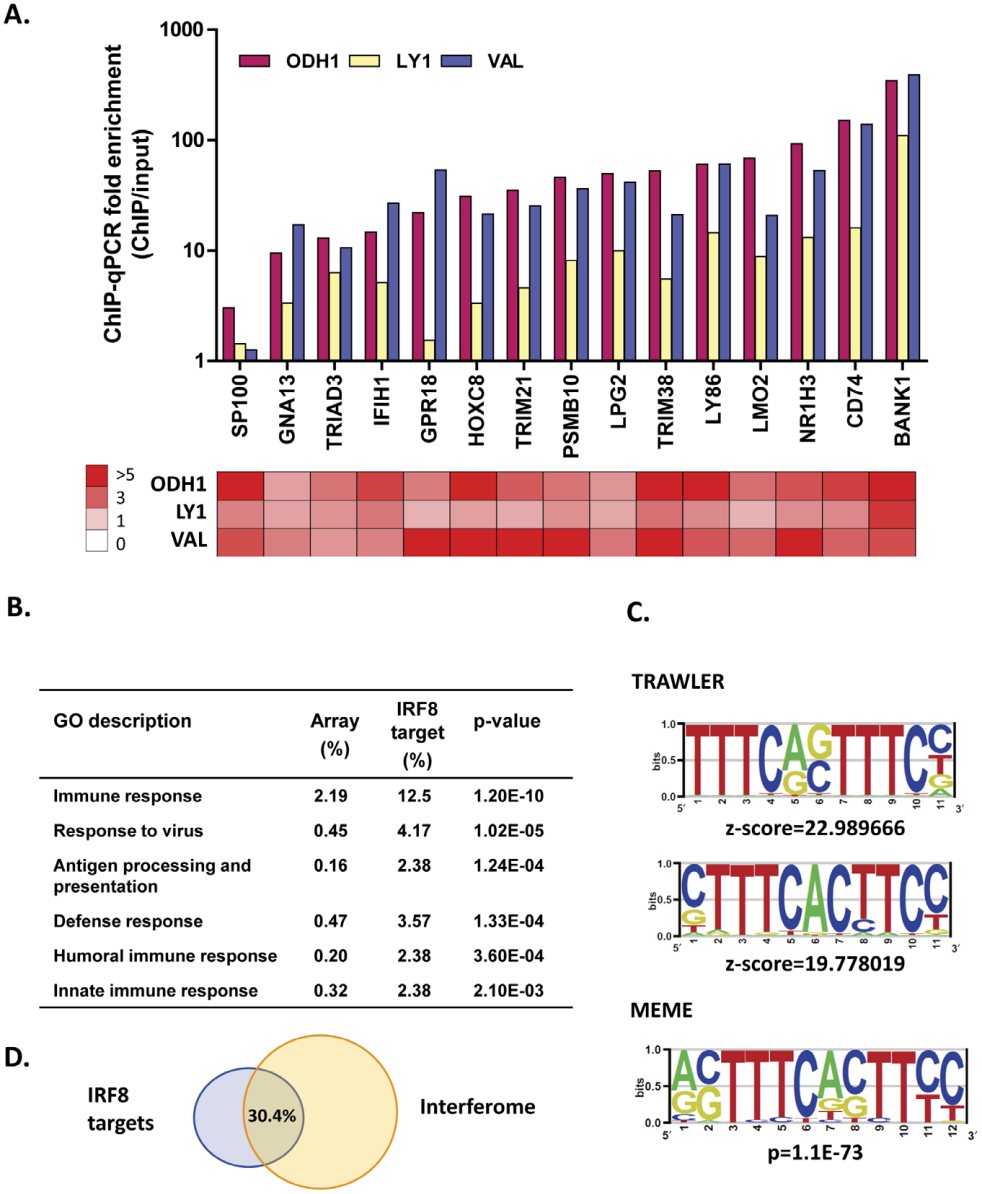
Validation of IRF8 ChIP-chip and functional classification of IRF8 targets (A) ChIP-qPCR validation using primer pairs surrounding the putative binding sites identified by ChIP-chip. For each locus, the fold enrichment comparing IRF8 ChIP DNA to input DNA is represented in the bar graph. The heatmap (lower panel) shows fold enrichment obtained from ChIP-chip. (B) Categorization of IRF8 targets by Gene Ontology (GO). Percents of genes in each category in the whole array or in the set of IRF8 targets are shown. p-values indicate significance of the enrichment for IRF8 targets in each GO category. (C) Motif analysis for IRF8 ChIP hits. Over-represented motifs were identified by TRAWLER and MEME. (D) A Venn diagram of IRF8 targets and interferon-responsive genes. The Interferome DB was used for identifying interferon-responsive genes.

An example of the fold enrichment of ChIP to input for each cell line is shown in Figure 1C in relation to the TSS for *TLR4* identifying a prominent peak directly over the TSS in all three biological replicates (FDR<1E−4) with no significant binding seen with MMS1. We then used ChIP-qPCR to validate the results of ChIP-chip binding assays for 15 genes identified in all three cell lines as targets for IRF8 by ChIP-chip (Figure 2A). Substantial enrichment was seen with most genes having at least 10-fold enrichment of IRF8 ChIP DNA compared to input DNA with ChIP material from ODH1 and VAL. The same general pattern was seen but with usually less enrichment with ChIP material from LY1. The basis for this cell line-specific difference is not understood**.** (Figure 2A, top). The heat map in the lower part of Figure 2A showing fold enrichment of IRF8 ChIP to input presented by ChIP-chip analysis demonstrated a high level of correlation between data obtained by ChIP-qPCR and ChIP-chip.

An examination of the genes identified as having IRF8 binding sites by ChIP-chip was performed by Gene Ontology (GO) analysis and revealed significant enrichments for immune response categories including innate and humoral responses, responses to virus as well as antigen processing and presentation (Figure 2B). The immune response category is comprised of 21 genes nearly half of which encode proteins involved in antigen presentation by MHC class I molecules (HLA–B, HLA–C, TAP1, TAP2, TAPBP, PSMB8, PSMB9) or MHC class II molecules (HLA–DRA, CD74, CIITA). Another large subset of genes encodes proteins involved in anti-viral responses (OAS1, OAS3, MX2, IFI35, IFIT3, IFIT3, IFIT5) or other aspects of IFN signaling (IRF9, BCAP31). The overlap with GO descriptions identified in similar ChIP-chip analyses of IRF8 target genes in myeloid cells is substantial [4], but is clearly and predictably demarcated by the category of humoral immune response. A seeming superimposition of B cell-specific and AID-dependent receptor diversification on the substrate provided by the classical innate immune functions of macrophages is in keeping with the suggestion of an earlier appearance of B than T cells in adaptive immunity, although other interpretations are possible [22].

To identify the characteristics of the cis-regulatory motifs over-represented in the set of IRF8-bound targets, repeat-masked ChIP sequences were queried in TRAWLER [23]. Two over-represented position weight matrices were generated by comparison to human 1000 bp upstream genome sequences as a background (Figure 2C). The top matrix (Z score = 22.99) contains two tandem canonical IRF binding sites (TTTC) separated by two nucleotides, characteristic of the IRF9/STAT1/STAT2 binding site termed an interferon stimulated response element (ISRE) [24]. The bottom matrix (Z score = 19.78) contains an IRF target sequence separated by two nucleotides from a TTCC motif that serves as a binding sequence for ETS family members including PU.1 (SPI1) [25]. This matrix closely resembles the previously identified TTTCNNTTCC motif, designated an ETS-IRF composite element (EICE) [1], [26]. MEME (Multiple EM for Motif Elicitation) is another widely used tool for searching for novel ‘signals’ in sets of biological sequences leading to discovery of new transcription factor binding sites. MEME analysis of the same data set identified a matrix (p = 1.1E–73) strikingly similar to the ISRE- and EICE-like motifs identified by TRAWLER (Figure 2C). We conclude that the DNA targets for IRF8 binding are divided among those that require heterodimerization with other IRF family or ETS family members. Although there are differences between the IRF8 binding sites identified by ChIP-chip in activated macrophages [4] and those defined here, they are basically very similar, reinforcing the concept of important commonalities between the transcriptional programs of macrophages and B cells.

Both IFNα/β and IFNγinitiate transcriptional activation of IFN-stimulated genes (ISGs) by activation of the JAK-STAT signaling pathways [24], [27], [28]. This results in binding of STATs as well as IRF and ETS family members to various IFN response elements including ISREs and EICEs described above. To examine the potential contributions of IRF8 to regulation of ISGs in GC B cells, we determined the proportion of IRF8 target genes that are part of the “Interferome” database of ISGs (http://www.interferome.org/; [29]; 30.4% of the IRF8 targets overlapped with the 1,996 genes in the Interferome database (Figure 2D, Table S2).

Taken together, the results of our ChIP-chip analyses of human GC-derived lymphoma cell lines identified over 250 target genes with binding sites located primarily at TSSs. The target sites were highly enriched for two distinct binding motifs very similar to canonical ISRE and EICE elements, respectively. A high proportion of the target genes were included in the Interferome of ISGs and were functionally involved in aspects of both innate and acquired immunity including antigen processing and presentation.

### Identification and characterization of IRF8 target sites in mouse lymphoma cell lines of GC origin

Our initial impetus for studying possible contributions of IRF8 to B cell development and function came from analyses of mouse B cell lineage lymphomas showing that levels of IRF8 expression varied significantly at progressive stages of differentiation. Expression was highest in diffuse large B cell lymphoma (DLBCL) of GC origin but was almost totally absent in tumors of mature plasma cells [17]. In that study, the IRF8-expressing NFS-202 cell line of GC B cell origin and IRF8 siRNA-expressing stable transfectants of that line were examined for selected gene expression by qPCR and for IRF8 target genes by ChIP. In the present study, we extended these analyses by examining these lines, two other IRF8-expressing cell lines of GC B cell origin (NFS-201 and NFS-205) and the IRF8-negative plasmacytoma cell line, MPC11, for gene expression profiling by microarray and for IRF8 and PU.1 target screening by ChIP-chip.

**Figure 3:**
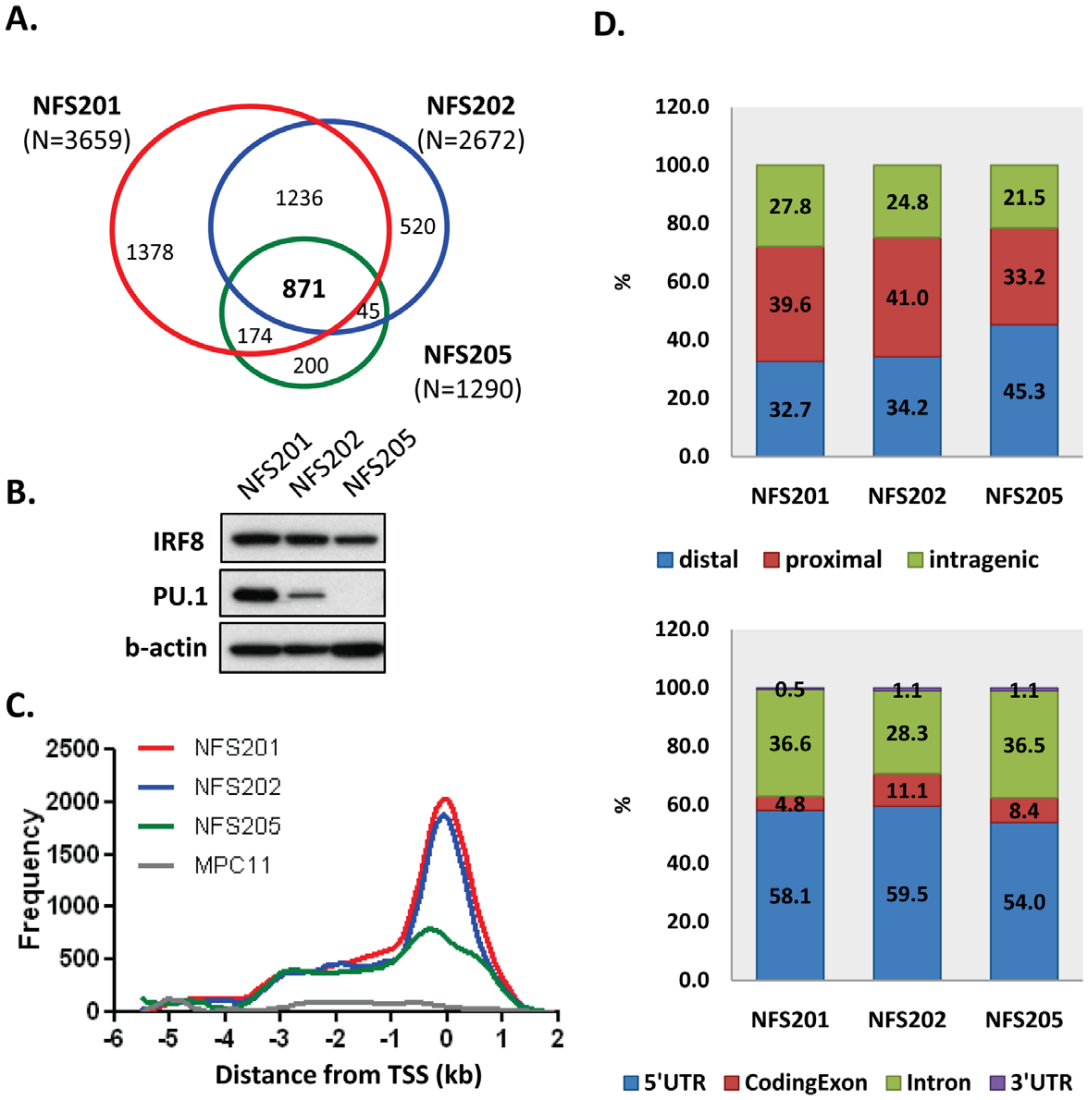
Identification of IRF8 and PU.1 targets in mouse cell lines of GC B cell origin. Labeled IRF8 ChIP samples and input samples were applied to Nimblegen MM8 385 k arrays and peak signals were identified by sliding window algorithm with threshold of FDR<0.01. (A) A Venn diagram for IRF8 targets. Numbers in parentheses indicate the number of IRF8 targets identified in each line. Venn diagram shows the number of IRF8 targets that belong to each area. (B) Western blots for IRF8 and PU.1 in the three cell lines. (C) Distribution of IRF8 ChIP hits by chromosomal location relative to transcription start sites (TSS). (D) Distribution of IRF8 binding related to the annotated structure of associated genes (top). The frequencies of IRF8 hits in the sub-structure of intragenic locations (bottom).

ChIP-chip analyses identified 3,659 and 2,672 IRF8 binding sites in NFS-201 and NFS-202, respectively, but only 1,290 sites in NFS-205 (Figure 3A). Among the targets, 871 were found to be common to all three lines (Table S3), a number 3.2-fold higher than for targets common to the three human cell lines. The reasons for this species-related difference are not clear but could be explained if the mouse lines were more similar to one another in differentiation state than the human lines or by the fact that all the mouse lines derive from a common NFS genetic background [30].

The lower number of IRF8 target sites identified in the NFS-205 cell line was also of interest. IRF8 differs from other members of the IRF family in that it can bind DNA only after heterodimerization with other members of the IRF family or with non-IRF transcription factors such as PU.1 [31], [32]. This prompted us to determine if PU.1 was expressed at comparable levels in the three cell lines. Unexpectedly, western blot analyses revealed significant differences among the lines for PU.1 expression while IRF8 levels were relatively similar (Figure 3B). PU.1 protein levels were high in NFS-201, substantially lower in NFS-202 and below the limits of detection in NFS-205.

Mapping of probes targeted by IRF8 in the three cell lines to the mouse genome paralleled studies of human IRF8 target sites with the majority mapping within 1 kb upstream or downstream of the TSSs of involved genes (Figure 3C). Interestingly, the proportion of sites mapping to this region was considerably lower for NFS-205 than the other cell lines, raising the possibility that a significant proportion of sites in the larger peaks documented for NFS-201 and NFS-202 may be targeted by IRF8/PU.1 heterodimers that tend to result in promoter activation [1]. In addition, a long shoulder of target sites in all three lines mapped from 1 kb to ∼4 kb 5′ to the TSSs, proportionally more than was seen with the human target sites. In contrast, no significant peaks were observed anywhere throughout this region with material prepared from the negative control cell line, MPC-11.

A more detailed characterization of the target sites for their localization to proximal or distal promoters and to intragenic regions is presented in Figure 3D. In the two PU.1-expressing cell lines, there was an enrichment for targets in proximal as compared to distal promoter regions (∼40% vs. ∼33%, respectively) with another ∼25% mapping as intragenic. Nearly 60% of the intragenic sites were localized to 5′ UTRs with almost none mapping to 3′ UTRs. Another third were intronic while less than 10% mapped to coding exons. Parallel studies of the PU.1-negative NFS-205 cell line revealed a predictable enrichment for targets mapping to distal promoter regions when compared to the PU.1-positive cell lines without much change in the proportions mapping to intergenic sites or their distributions among subsets of these sites (Figure 3D).

These results indicated that genes targeted by IRF8 in germinal center cells of both humans and mice are most often characterized by binding sites in proximity to TSSs while also suggesting that the differential distribution between proximal and distal promoter regions is likely to be influenced by the availability of PU.1 as a partner protein.

### Expression profiling of IRF8 regulated genes

**Figure 4:**
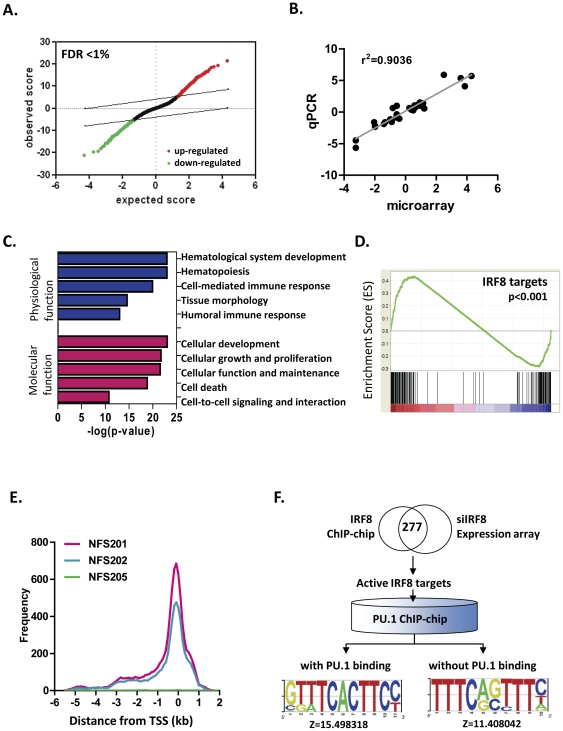
Transciptome analysis of mouse DLBCL cell lines stably expressing siIRF8. Total RNAs from NFS-202 cell lines stably expressing siIRF8 and control cell lines were applied to NIAID mouse expression arrays. (A) Significant Analysis of Microarray (SAM) plots for identification of differentially expressed genes in knock-down cell lines. Both up- and down-regulated genes were identified with FDR<0.01. (B) qPCR validation of differentially expressed genes from microarray analysis. Fold change of siIRF8 cell lines vs. control cell lines were plotted against fold change in microarray. Values are in log2 scale. Linear regression analysis was performed (p<0.0001). (C) Functional classification of significant genes in IRF8 knock-down cell lines. Fisher's exact test was performed to identify significantly enriched biological categories using Ingenuity Pathway Analysis (IPA). Log-transformed p-values from Fisher's exact test are shown on the x-axis. (D) GSEA analysis of mRNA expression profiles for IRF8 knock-down cells vs. control cells. Relative expression was rank-ordered by fold change of five replicate IRF8 knock-down samples vs. five replicate control samples. Genes associated with IRF8 binding sites (IRF8 ChIP targets) were strongly correlated with IRF8 expression level. The color bar at the bottom indicates up-regulated (red) and down-regulated (blue) genes. (E) Distribution of PU.1 ChIP hits by chromosomal location relative to transcription start sites (TSS). PU.1 ChIP-chip analyses were done using NFS-201 and NFS-202 cells that express PU.1 and NFS-205 cells that are PU.1-negative. Labeled PU.1 ChIP samples and input samples were applied to Nimblegen MM8 385 k arrays and peak signals were analyzed by sliding window algorithm with threshold of FDR<0.01. (F) Classification of mouse IRF8 targets with altered expression by siIRF8 in relation to PU.1 binding to the gene. A Venn diagram of genes bound by IRF8 from ChIP-chip and genes that were significantly altered in IRF8 knock-down cells as indicated by gene expression microarray (false discovery rate<0.01). Those IRF8 targets with differential expression were classified into two groups based upon the observation of PU.1 binding from ChIP-chip. The top over-represented motifs were identified in two groups of IRF8 ChIP-hits by TRAWLER using mouse 1 kb promoter set as background.

To identify genes affected at the transcriptional level by alterations in IRF8 expression, we performed gene expression profiling of NFS-202 cells stably transfected with an IRF8-specific or a control siRNA [17]. Using the Significance Analysis of Microarray (SAM) tool, we identified 954 down-regulated genes and 1107 up-regulated genes in IRF8 knockdown cells (Figure 4A, Table S4), consistent with the activities of IRF8 as both a transcriptional activator and a transcriptional repressor. To validate the transcriptional effects of IRF8 suppression identified by microarray analyses, we quantified expression of 29 affected genes that were present on a commercial qPCR array. There were strong correlations between the expression levels of genes determined by either approach (1/slope  = 0.74, r^2^ = 0.90) (Figure 4B).

A gene ontology (GO) assessment of genes affected transcriptionally by downregulation of Irf8 revealed enriched gene clusters associated with a variety of cellular processes centered on hematopoietic differentiation as well as cell-mediated and humoral immune responses (Figure 4C, top panel). Molecular functions affected most prominently by altered IRF8 expression were those involved in cell development, growth and proliferation, but also cell death and cell-to–cell signaling (Figure 4C, bottom panel).

We next applied Gene Set Enrichment Analysis (GSEA) to determine if the expression of genes identified as targets of IRF8 binding by ChIP-chip was altered by suppressing IRF8 expression in NFS-202 cells (Figure 4D). The results showed that IRF8 target genes were significantly enriched in either up-regulated genes or down-regulated genes in the IRF8 knock down cell line with the bottom part of enrichment plot showing where IRF8 targets appear in the ranked list of genes in the expression array.

### Relationship of PU.1 binding to subsets of IRF8 target genes

PU.1 is a key transcription factor required for the development of all hematopoietic cells [33], for lineage fate decisions leading to B cell and macrophage differentiation [34], and for effector functions of mature macrophages. Several recent studies are also suggestive of roles for PU.1 in differential distribution of B cells into the splenic follicular and marginal zone compartments [35] and as a negative regulator of late B cell differentiation [36], although specific target genes have not been identified. Prior studies demonstrated that both PU.1 and IRF8 are recruited to DNA sequences defined as EIREs (ETS/IRF response elements) or EICEs (ETS/IRF composite elements) that lead to transcriptional activation [1], [37]. The fact that the number of IRF8 target sites was significantly reduced in PU.1-deficient NFS-205 cells suggested that a significant number of germinal center B cell genes may be targeted by PU.1/IRF8 heterocomplexes.

To examine this possibility, we first used ChIP-chip analyses to characterize PU.1 target sites in NFS-201 and NFS-202 cells with NFS-205 serving as a negative control and identified 1,764 target sites common to both IRF8-expressing cell lines (Table S5). As for IRF8 targeted locations, the great majority of target sites for both lines mapped within 1 kb 5′ to 1 kb 3′ to the TSSs of involved genes (Figure 4E). Interestingly, ∼75% of the genes identified as targets of PU.1 in GC B cells were distinct from those identified previously by ChIP-chip analyses of PU.1 targets in the macrophage cell line RAW264.7 [38] (Figure S1). After eliminating false positive targets for IRF8, defined as those found in the MPC-11 control cells, and those for PU.1, defined as targets identified in the PU.1-negative NFS-205 cells, we identified 355 genes commonly targeted by both transcription factors.

By combining ChIP-chip and gene expression microarray studies of the mouse cell lines, we identified 277 genes that were targeted by IRF8 and that were significantly altered in expression in siIRF8 knockdown cells (Figure 4F). The IRF8 targets were then segregated into two clusters based on whether they were also targeted by PU.1. The results of these studies revealed a near 50∶50 split among IRF8 target genes for those that were also targeted by PU.1 and those that were not. We then used the Trawler algorithm to characterize binding motifs associated with targets bound by IRF8 alone and those targeted by the presumed heterodimers. Predictably, the canonical ISRE motif - GAAANNGAAA (TTTC A/G G/C TTTC) - was identified as the top matrix in the subset of IRF8-only targets (Figure 4F; Z = 11.4). Similar analyses of the sites targeted by both IRF8 and PU.1 identified the sequence GTTTCACTTCC (GGAAGTGAAAC), identical to EICE elements, as the most over-represented motif (Z = 15.5).

A broader picture of the transcriptional landscape governed by IRF8 in mouse B cells is presented in Table 1 in which IRF8 target genes, categorized functionally, are further annotated for the effects of IRF8-specific siRNA on gene expression and ChIP-chip analysis of PU.1 binding. IRF8 target genes were associated with a wide spectrum of biologic processes, including components of innate and adaptive immunity, as highlighted previously for human targets. In addition, substantial numbers of genes were associated with the categories of GTP signaling, transcription factors, cell adhesion, as well as secondary protein modifications by ubiquitylation, SUMOylation and ADP ribosylation. ChIP-chip studies showed that, as noted above, nearly half of the IRF targets were also targeted by PU.1, and gene expression studies indicated that IRF8 was directly involved in the expression of ∼60% of the targeted genes. These results indicated that IRF8 is involved in broader aspects of B cell biology than was appreciated previously and that there is significant overlap between genes regulated transcriptionally by IRF8 in B cells and those targeted in macrophages or dendritic cells as reported by others [4], [10], [20], [21].

**Table 1:** Functional and transcriptional features of IRF8 target genes in mouse cell lines of GC B cells.

	Activated	Repressed	Not determined
**Adaptive immunity**	***B2m***, *C2ta*, Erap1, *H2-DMb1,* H2-Ea, H2-M3, *H2-Q7, H2-Q8* Igl-V1, Rfx5, **Tap1**, **Tap2**	*Bcap31*, ***Blnk***, ***Btk***, Cd274, *Cd52*, Cd74, , *Ms4a1 (CD20),* Sla	*Cd40*, *Cd69*, *Dapp1*, *H2-D1, H2-DMb2*, *H2-K1*, H2-K3, *H2-Q1*, *H2-Q5*, H2-T10, H2-T17, H2-T22, H2-T9, *Tapbpl*
**Innate immunity**	Ifi35, **Irf4**, Mov10, *Mx2*, Zbp1	**Ifih1, ** ***Irf5***, *Isg20, Isgf3g*, Ly86, *Ncf4*, *Nosip*, Oas1c, Oasl2, *Tlr4, Tlr9*	Crry, Ddx58, *Hmgb1*, Ifit1, *Igtp*, *Iigp2*, Il12rb1, *Irf2*, Irgm, Invs1abp, Oas1b, Tbk1, Tff1, *Ticam2*, Tlr12, *Tlr6,* Trim21, *Zc3hav1*
**Chemokines, cytokines, receptors**	**Ccl5**, Ccl6, Csprs, Epha2, Grina, Il12rb1, Mst1, **Ptch1**, **Socs1**, **Tnfsf10**, ***Traf1***	Aif1, Arnt, *Btc*, Ccrl2, Entpd1, Ltbp1, S100a13, Spred2, Tbgr1, Tnfrsf13b, **Tnfrsf1b**	Arts1, Blr1, *Cxcl0*, Il28ra, *Phf11*
**DNA repair**	Gadd45g, Hus1, Parp9, Rad51l1, *Shfm1*	Bard1, ***Brca1***, *Ercc6l*, *Pold4*, ***Xrcc5***	*Dclre1c*, Top3a, Trp53
**Apoptosis**	*Apcs*, *Casp1*, **Cflar,** Thyn1, *Traf1*	*Casp8ap2*, *Emp3*, *Pdcd11*, *Prdx6*	*Bcl2a1a, Bcl2a1c, Bcl2a1d*, Bid, *Birc1f, Birc1g*, Birc2, *Casp9*, Tmbim1, Trp53
**GTP signaling**	Cysltr2, Gem, Gnaz, *Rabggta*, *Rhobtb1*, Sar1b	*F2rl1*, *Gbp4*, *Igtp*, *Khdrbs1*, *Rab2b*, *Rabgap1l*, Rhebl1, Rufy3	*Arhgap25*, Arhgap30, *Fgd2*, Gbp1, Gbp2, Gbp5, *Gimap9*, *Gnb2*, Gnl31, *Gpr18*, Gprk5, *Lsg1*, *Rab11b*, *Rab21*, Rab3ip, *Rab8b*, Rapgef6, *Rkhd2*, *RP23-336J1.4*, Tagap, Tgpt, *Ubxd5*, Usp6nl
**Phosphatase**		*Ppm1k*, *Ptpn18*, **Ptprc**	*Dusp2*, Ppfibp2, *Ppm1m*, *Ppp1r11*, Ppp1r15b, *Ptprj*
**Protein kinases**	Akap13, Fgfr1op2, **Stat1**	Aurkb, *Madd*, Miki, *Skil*, Spred2, Tbk1	*Btk*, Csnk1a1, Flt3l, Prkrip1, Prkrir, Ptk2, *Raf1*, Socs1, Stat2, Ttk, *Ywhag*
**Cell cycle regulation, cell division**	*Ccng2*	**Cdkn2c**, *Cenpa*, Mad1l1, Mad2l2, *Tob2*, Zwilch	Cdca1, Rnf123, Tbrg1, Trp53
**Transcription factor, transcriptional regulation**	*Eef1b2*, Elf4, Mybl1, Nap1l3, Notch1, Tcfap2a	Armcx3, *Atxn7l1*, *Bcl9l*, *Bcor*, *Cited2*, *Crem*, *Elf1*, **Ets1**, **Irf7**, *Mnt*, Mrcs2, ***Nfkb1***, Nmi, Pcgf5, *Prrg2*, Rnf141, *Skil*, Suv39h1, *Zbtb32*, *Zfp422*	Baz2b, Gtf3c5, *Irf2*, *Irf5*, Mzf1 (Zfp98), Rbbp9, *Rfx4*, Rfx5, *Sin3a*, *Sirt6*, Tcea1, Tcof1, *Usf1*, *Zfp143*, *Zfx*, Zmynd11
**Adhesion, extracellular matrix**	*Cbln3*, *Cd37*, Epsti1, Itgb1, Pcdhgc4, Pcdhgc5	*Cd53*, Lgals8, *Lpxn*, Mgat5, Pcdhgb4, *Pecam1*	Cd164, Clec1a, *Clec12a*, Itgb3bp, *Pdlim2*
**Cytoskeleton**	*Clasp1*, *Elmo1*, Exoc2, *Inadl*	Ehd2, Ide, Katna1, *Lsp1*	Clasp10, Marcks, Mast3, *Ptk9l (Twf2),* Tpt1, Tuba2, Tuba7, *Tubb3*
**Protease, proteasome, autophagy**	Edem1, *Nbr1*, Psma2, **Psmb10**, *Psmb8*, Psmb9	*Ncstn*	Ctrl, Ctso, *Ctss*, *Psme2*, *Uvrag*
**RNA processing, transcription, translation**	Cstf2t, *Eif4a2*, Erh, Prkrir	Cpsf2, *Prkrip1*, Qtrtd1, Sf3a3, Trove2, *Wibg*	Ddx21, Ell3, *Pold4*, Rnase4, Rexo4, *Sf1*, *Tsen54*, Xrn1, Zcchc6
**Ubiquitylation, SUMOylation, ADP ribosylation**	*Nedd4*, Rnf123, Trim21, Ube1l, Ube2i, Ubl7	*Arl6ip1*, Fbxo8, Mtbp, *Parp8*, *Trim2*5, *Trim30*, Uchl5, Usp18, Usp52, *Zfp91*	Fbxo17, Fbxo36, Fbxo39, Fbxo43, Parp14, Parp9, *Rnf31*, Trim41, Ube2v1, Ufc1, Usp14, Usp44
**Protein processing, chaperones**	Cct6b, *Dnaja2*, *Dnajc10*, *Ppil3*, Tmed7	*Arsk*, *Ebag9*, ***Lrmp***, Nvl2	
**Solute transporter**	*Osbpl3*	Cutc, *Slc37a2*	
**Ion channel**	*Kcnq5*		
**Mitochondria**	Cyp4v3, Fars2, Mrpl32	Cap1, Mrpl3, Mrpl13, Oxsm	
**Fatty acid, cholesterol metabolism**	Acad8, Plscr1, *Stoml1*		

364 IRF8 targets with altered expression by siIRF8 were classified to their functional involvement in GC B cells (only known genes were listed). Genes in **bold** were confirmed by qPCR; in *italics* are both IRF8/PU.1 targets.

### IRF8 network common to human and mouse B cells

**Figure 5:**
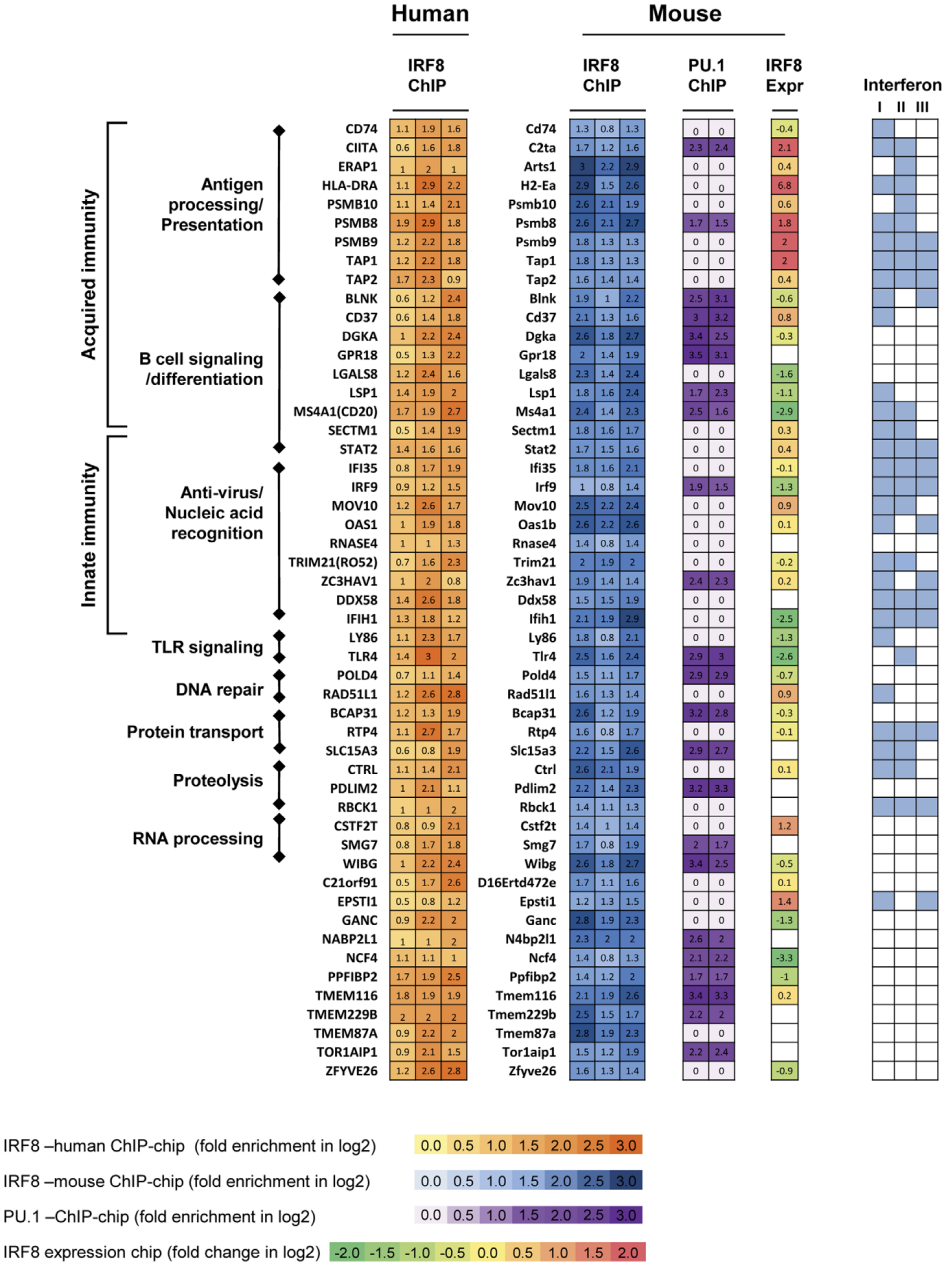
IRF8 targets common in human and mouse. IRF8 targets common in both human and mouse were identified by intersecting human and mouse ChIP-chip analysis. These 51 targets are listed along with data on fold enrichment of IRF8 ChIP vs. input. Data obtained from human cell lines LY1, ODH1 and VAL are shown in the left most heatmap. IRF8 ChIP-chip data from mouse cell lines NFS-201, NFS-202 and NFS-205 are shown in blue. Fold enrichment of PU.1 ChIP vs. input from NFS-201 and NFS-202 are shown in purple. IRF8 expression (Expr) column indicates expression levels of genes identified as activated or repressed by IRF8. Reported responsiveness of genes to interferon type I, II, or III is shown in pale blue in the rightmost map. Numbers in ChIP-chip data are fold enrichment and those in expression array are fold change of control vs. siIRF8 cell lines.

Comparisons of genome-wide transcription factor binding patterns across species indicate that a large proportion of enhancers are species specific with significant divergences between human and mouse [39], [40]. Our analyses of both mouse and human cell lines of GC B cell origin allowed us to rephrase this issue in terms of IRF8 targets in B cells. Using stringent criteria, we identified 51 genes that were targeted by IRF8 in the cell lines of both humans and mice, with further analyses demonstrating that 45% were also targeted by PU.1 (Figure 5). Among the 51 genes, 41 were represented on the expression arrays used for transcriptional profiling of NFS-202 IRF8 knockdown cells, making it possible to determine the relationships between target occupancy and regulation of gene expression. Significant changes in transcript levels detected for 27 of the 41 genes implied that 15 were activated and 12 were repressed by IRF8 or IRF8 plus PU.1. Functionally, over half of the targeted genes common to humans and mice were readily identified as contributing to various aspects of acquired and innate immunity with B cell signaling and differentiation, antigen processing and presentation, anti-viral activities and nucleic acid recognition being most prominent. Importantly, 90% of these “immune” genes were previously shown to be responsive to stimulation with type I, type II or type III IFNs and, not infrequently, all three (Figure 5).

**Figure 6:**
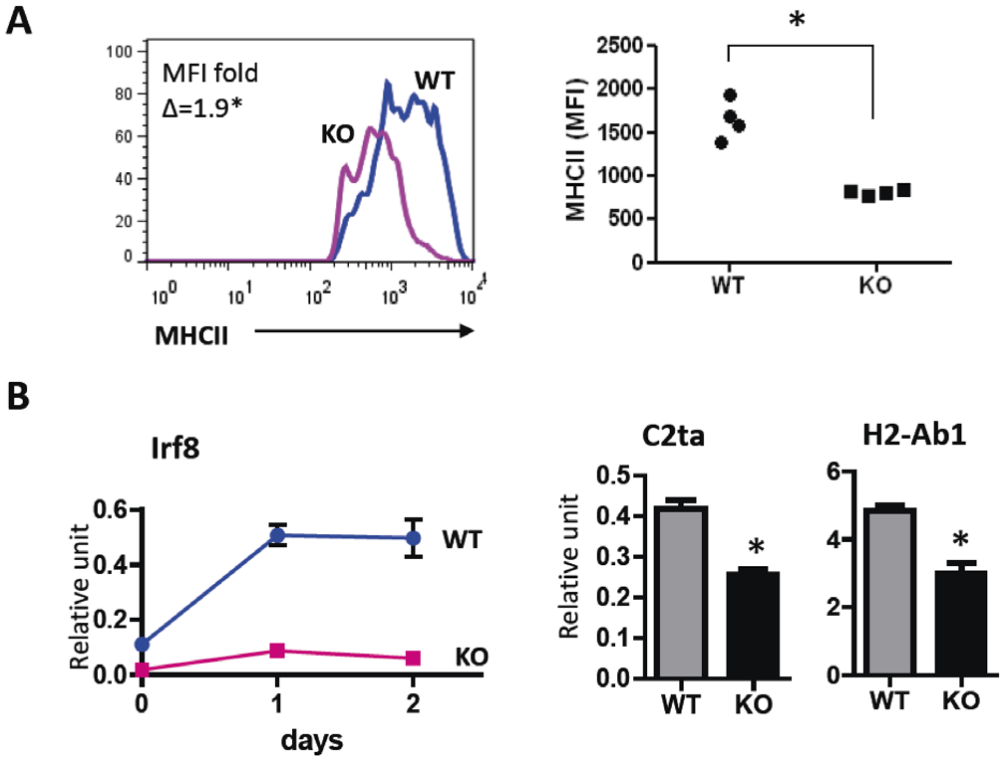
MHC II expression in germinal center B cells of IRF8 knock out mice. (A) MHC class II expression in GC B cells in IRF8 KO and WT mice. Representative histogram for MHC class II expression in GC B cells (left panel). Dot plot shows significant difference in MHCII expression between two groups. *, p<0.05 from Mann-Whitney test. (B) MHC class II expression in *in vitro* stimulated B cells. Purified B cells were stimulated with IFNγ for 2days. Transcript level of *Irf8* were determined by qPCR at days 1 and 2 after stimulation (left panel). Transcript levels of *C2ta* and *H2-Ab1* in wt and KO B cells determined by qPCR are shown (right panel). Data from four different mice in each group were examined by t-test. *, p<0.05.

These observations prompted us to validate this network by studying expression levels of MHC class II genes and CIITA in B220 gated FAS^+^GL7^+^ GC B cells of IRF8 conventional KO and control mice. First, spleen cells from these mice were analyzed by flow cytometry for the levels of the MHC class II expression on GC B cells. Flow cytometric studies showed that the levels of MHCII expressed by GC cells of IRF8 KO mice were significantly lower than for cells of wt mice (MFI fold change  = 1.9, Figure 6A). We also quantified transcript levels for *C2ta*, the master control factor for expression of MHC class II genes and one of the class II genes, *H2-Ab1*, following stimulation of WT and IRF8 KO B cells with IFNγ. The results showed that both genes were expressed at significantly lower levels in stimulated KO cells (p<0.05) (Figure 6B).

The results of this study provide the first comprehensive picture of the transcriptional programs and cellular pathways governed by IRF8 in mature B lineage cells as viewed through the lenses provided by analyses of cell lines of GC origin from humans and mice. Our conclusions derive from a synthesis of data from ChIP-chip analyses of IRF8 occupancy of target sites in both species, microarray-based transcriptional profiling of the mouse cell lines, and ChIP-chip analyses of PU.1 target sites in the mouse cells. The findings indicate that IRF8 is involved in the regulation of a large number of genes of known importance to various aspects of the biology of mature B cells.

**Figure 7:**
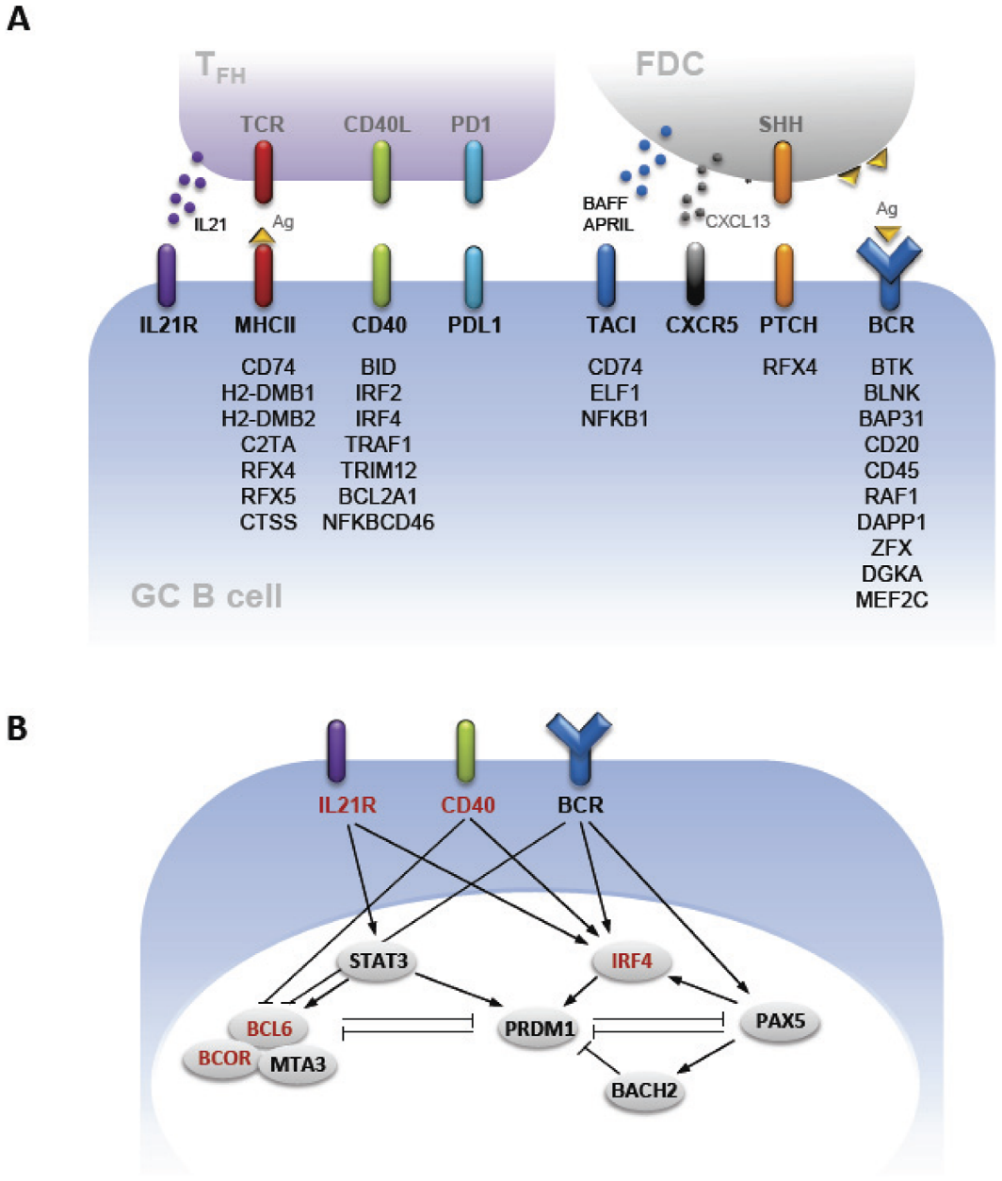
The roles of IRF8 in GC B cells. IRF8 targets in B cells were presented in context of signaling pathways in GC.(A) Cross-talk between B cells and T cells and follicular DC in GC. IRF8 targets in B cells are shown together with ligands or products from TFH or FDC cells. (B) Signaling downstream of IL21R, CD40, and BCR. IRF8 targets are shown in red.

As illustrated in Figure 7A, these targets include critical components of the molecular crosstalk among the specialized cell types that comprise the GC reaction – GC B cells, T_FH_, and follicular dendritic cells (FDC). Communications between GC B cells and T_FH_ are mediated by a series of molecular pairings that include the cytokine IL21 and its receptor, IL21R, the T cell receptor and antigen presented my MHC Class II molecules, CD40 and its ligand, CD40L, and PDL1 and its receptor, PD1. These couplings promote enhanced secretion of IL21 by T_FH_, driving the generation of both memory B cells and plasma cells (reviewed in [41]). Brief encounters of antigen-specific B cells with antigen present on the surface of FDC combine with survival signals provided by T_FH,_ through engagement of PDL1, by FDC secretion of BAFF and APRIL, ligands for TACI, and CXCL13, the ligand for CXCR5, and by pairing of Sonic hedgehog (SHH) on FDC with Patched (PTCH) to promote positive selection, affinity maturation and clonal expansion of GC B cells. Some of the IRF8 target genes that either promote the expression and activity of the B cell receptor/ligand pairs or contribute to their downstream signaling pathways are listed under the different cell surface components.

Although not illustrated here, there are a large number of cell membrane, cytosolic and endosomal proteins encoded by IRF8 targeted genes that function as sensors of pathogen-associated molecular patterns. It is increasingly well recognized that when engaged, these molecules can exert major influences on B cell activation induced by BCR ligation or signaling through other receptors [42], [43], [44], [45]. While crosstalk between BCR and TLR signaling thus contributes to normal responses to both T-dependent and T-independent antigens, it is also clear that aberrant activation of these signaling molecules can promote the development of profound humoral autoimmunity [46], [47], [48]. Modulation of gene expression by IRF8 may thus contribute to the balance between physiologic and pathoglogic B cell reactivity.

The molecular transitions required for the maturation of GC B cells to plasma cells are governed by a relatively small set of transcription factors that lie downstream of signals generated by engagement of the IL21R, CD40 and the BCR (Figure 7B). B cell identity is promoted by BCL6, PAX5 and BACH2, which suppress transcription of PRDM1, in opposition to the drive for plasma cell maturation advanced by XBP1 (not shown) and PRDM1, which in turn suppresses PAX5 and BCL6. The contributions of IRF4 to this scheme are complex and incompletely understood as it is required for the differentiation and function of mature B cells as well as plasma cells. The results of this study showed that many of the components of this network are transcriptional targets for IRF8 (Figure 7B).

Our systemic and comprehensive approaches have elucidated the roles played by IRF8 in governing transcriptional network in GC B cells. However, understanding the full nature of IRF8 contributions to B cell biology from the earliest stages of lineage commitment to terminal differentiation will require more detailed investigations of the partnering of IRF8 with other proteins at its target sites. As noted previously, IRF8 can bind DNA only after heterodimerization with other transcription factors. While our studies demonstrated that IRF8 associates with PU.1 at about half of the target sites defined in GC B cells, the full picture of IRF8 bound to these sites may be even more complex as IRF8 has been shown to physically associate with both PU.1 and IRF4 to regulate gene expression through recognition of ISRE and EICE sequence elements [1], [49].

## Materials and Methods

### Cell lines and mice

Human lymphoma cell lines of GC origin - LY1, ODH1, and VAL - were kindly provided by Dr. Riccardo Dalla-Favera (Columbia University). LY1 and VALB are GC B cell type DLBCL. ODH1 is a Burkitt lymphoma cell line of type I latency for EBV infection**.** The MM cell line MMS1 was provided by Dr. Michael Kuehl, National Cancer Institute, NIH and mouse plasmacytomas cell line MPC-11 was from Dr. Michael Potter, National Cancer Institute, NIH. Mouse cell lines of GC origin - NFS-201, NFS-202 and NFS-205 - were generated in our laboratory. NFS-201 and NFS-205 derived from DLBCL tumors of centroblastic and immunoblastic subsets [50], respectively, and NFS-202 from the scid transplant of a follicular B cell lymphma. NFS-202 IRF8 siRNA stable transfectant was described previously [17]. IRF8 KO mice [6] and littermate controls were studied at six to ten wk of age. Animal studies were performed under NIAID IACUC approved protocol LIP-6.

### Chromatin immunoprecipitation (ChIP)-chip

ChIP was performed according to the manufacturer's protocol (Nimblegen, Reykjavík, Iceland). Briefly, cell lines were cross-linked with formaldehyde and the chromatin extracts were sonicated (Misonix Sonicater 3000). Following immunoprecipitation with anti- IRF8 antibody (sc-6058x, Santa Cruz Biotechnology), DNAs were purified from ChIP samples and input control samples. Purified DNAs were blunt ended, ligated with linkers and amplified by PCR. Amplified ChIP samples and input DNA samples were labeled with Cy3 and Cy5, respectively. Labeled samples were pooled and hybridized onto HG18 385 k two array set for human samples and MM8 385 k two array set for mouse samples. ∼60,000 transcripts and ∼48,000 transcript were represented in the human promoter arrays and mouse promoter arrays, respectively. Arrays were scanned (NimbleGene MS 200). Peaks were detected by searching for four or more probes with signals above the cutoff value, which was a hypothetical maximum, mean+6SD, using a 500 bp sliding window. False discovery rate (FDR) score was calculated with 20 times randomization (GSE30356 in GEO). ChIP targets were functionally classified based on GO (http://geneontology.org) and the significance of the enrichment for the ChIP targets in each category was examined in GeneMerge (http://genemerge.cbcb.umd.edu).

### Motif search

Genomic sequences were retrieved from the UCSC databases for HG18 and MM8. Over-represented motifs were analyzed using Trawler in EMBL (http://ani.embl.de/trawler) and MEME (http://meme.nbcr.net).

### ChIP- Quantitative Real Time PCR (qPCR)

ChIP samples were also analyzed by qPCR. Primer designs were based on IRF8 binding sequences from the ChIP-chip data; primer sequences are listed in Table S1. The results are presented as the fold-enrichment over input.

### Transcriptional profiling

Total RNAs prepared from six replicate samples of NFS-202 cell lines stably transfected with IRF8 knockdown siRNAs [17] and control vector transfected cell lines were applied to NIAID-mouse gene expression arrays. Scanned images were analyzed as detailed previously [51] and raw data were normalized with LIMMA package in R (www.r-project.org). Differentially expressed genes were identified with SAM (Significance Analysis of Microarrays; www-stat.stanford.edu/∼tib/SAM) with 1% FDR. Significant genes were divided into their functional categories and tested for significance of those enrichments in IPA (Ingenuity Pathway Analysis). The enrichment of IRF8 ChIP targets identified by ChIP-chip in this expression profiling was also examined by gene sequence enrichment analysis (GSEA) (http://www.broadinstitute.org/gsea/index.jsp) (GSE30356 in GEO).

### qPCR

The methods used for qPCR were described previously [51]. RNA was prepared from cell lines and activated normal B cells using the RNeasy mini kit (Qiagen) and the quality of the RNA was examined by Bioanalyzer (Agilent). cDNA was synthesized according to the manufacturer's protocol (MessageSensor RT kit, Ambion). cDNA was applied to 384-well mouse B cell qPCR plates (Bar Harbor BioTechnology) and the reaction was carried out using an ABI PRISM 7900 HT sequence detection system (Applied Biosystems). All experiments were done in triplicate. The correlations between gene expression as determined by microarray and qPCR were tested with linear regression.

### Western blotting

Western blotting was performed as described previously [51] using antibodies to IRF8, PU.1 and β-actin (Santa Cruz).

### Flow cytometry

Cells were prepared and stained as previously reported [51] using monoclonal antibodies specific for B220, FAS, GL7, and MHCII I-A(b) purchased from BD Pharmingen. Flow cytometric analyses were performed on a FACSCalibur (BD). Data were analyzed by FlowJo (BD).

### B cell activation

Splenic B cells were purified with DynalBeads (Invitrogen) and cultured at 1×10^6^/mL in 24 well plates with RPMI media containing 10% FBS and 1% penicillin/streptomycin at 37^°^C. Cells were activated with IFNγ (500 ng/ml) for 2 days. Total RNAs were extracted and transcript levels of MHCII and C2ta were measured by qPCR.

## Supporting Information

Figure S1 **Comparison of PU.1 targets in B cell with PU.1 targets in macrophage cell lines.** Comparison of PU.1 targets in B cell with PU.1 targets in macrophage cell lines (GSE9011 in GEO). A Venn diagram (left) shows partial overlap between B cell and macrophage targets in addition to B cell- specific or macrophage-specific targets. Fold enrichment of PU.1 ChIP to input in B cells were plotted against fold enrichment observed in macrophage ChIP-chip (right).
(TIF)

Table S1 **List of primers for ChIP-qPCR.**
(XLSX)

Table S2 **List of direct IRF8 targets in human GC B cell lines.** FDR(false discovery rate) is <0.05. Score is the fold enrichment of ChIP to input in log2.
(XLSX)

Table S3 **List of direct IRF8 targets in mouse GC B cell lines.** FDR(false discovery rate) is <0.01. Score is the fold enrichment of ChIP to input in log2.
(XLSX)

Table S4 **List of mouse IRF8 targets with significant changes in their expression in IRF8 knock down cells.** 1% of q-vaule( = FDR) in SAM was used to identify differentially expressed genes in IRF8 knock down cells.
(XLSX)

Table S5 **List of PU.1 targets in mouse GC cell lines.** FDR(false discovery rate) is <0.01. Score is the fold enrichment of ChIP to input in log2.
(XLSX)
