# Supplementary figures and images for: IRF8 Governs Expression of Genes Involved in Innate and Adaptive Immunity in Human and Mouse Germinal Center B Cells
Source: PLoS One. 2011 Nov 11;6(11):e27384. doi: 10.1371/journal.pone.0027384 (PMC3214047; doi:10.1371/journal.pone.0027384)

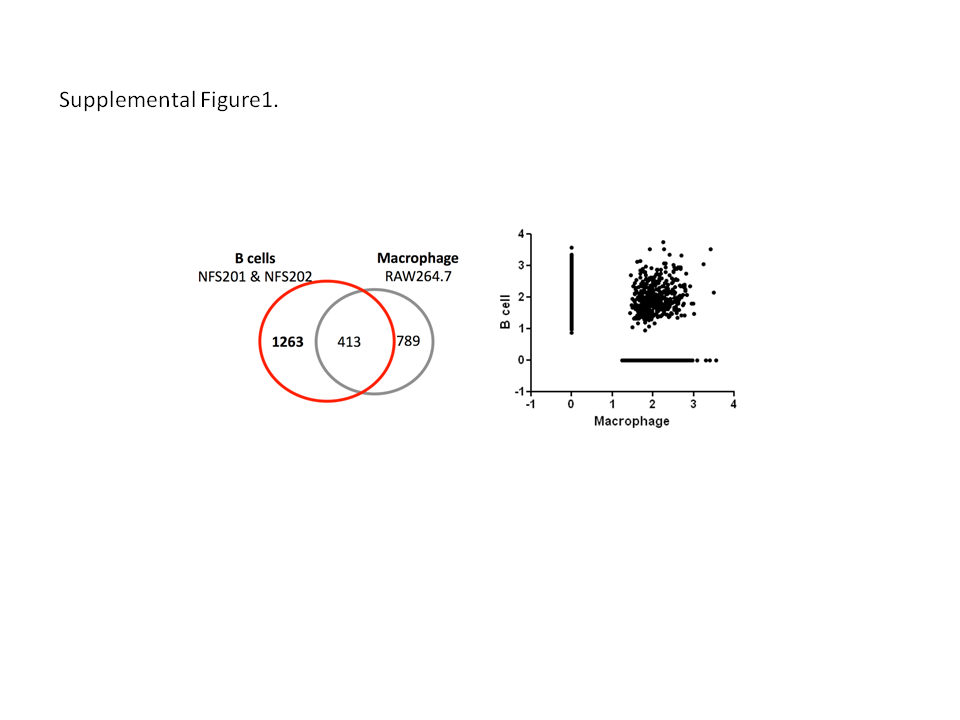

Supplement: Figure S1 — Comparison of PU.1 targets in B cell with PU.1 targets in macrophage cell lines. Comparison of PU.1 targets in B cell with PU.1 targets in macrophage cell lines (GSE9011 in GEO). A Venn diagram (left) shows partial overlap between B cell and macrophage targets in addition to B cell- specific or macrophage-specific targets. Fold enrichment of PU.1 ChIP to input in B cells were plotted against fold enrichment observed in macrophage ChIP-chip (right). (TIF) [file pone.0027384.s001.tif]
